# Chemokine Receptor CCR2b Enhanced Anti-tumor Function of Chimeric Antigen Receptor T Cells Targeting Mesothelin in a Non-small-cell Lung Carcinoma Model

**DOI:** 10.3389/fimmu.2021.628906

**Published:** 2021-03-11

**Authors:** Yanan Wang, Jing Wang, Xinyi Yang, Jinlong Yang, Panpan Lu, Lin Zhao, Bokang Li, Hanyu Pan, Zhengtao Jiang, Xiaoting Shen, Zhiming Liang, Yue Liang, Huanzhang Zhu

**Affiliations:** State Key Laboratory of Genetic Engineering and Engineering Research Center of Gene Technology, Ministry of Education, Institute of Genetics, School of Life Sciences, Fudan University, Shanghai, China

**Keywords:** CAR T cell therapy, CCR2b, MCP-1, migration, NSCLC, mesothelin, chemokine, chemokine receptor

## Abstract

Chimeric antigen receptor (CAR) T cell therapy faces a number of challenges for the treatment of non-small-cell lung carcinoma (NSCLC), and efficient migration of circulating CAR T cells plays an important role in anti-tumor activity. In this study, a CAR specific for tumor antigen mesothelin (Msln-CAR) was co-expressed with cell chemokine receptors CCR2b or CCR4. Findings showed that CCR2b and CCR4 enhanced the migration of Msln-CAR T cell *in vitro* by transwell assay. When incubated with mesothelin-positive tumor cells, Msln-CCR2b-CAR and Msln-CCR4-CAR T cell specifically exerted potent cytotoxicity and produced high levels of proinflammatory cytokines, including IL-2, IFN-γ, and TNF-α. Furthermore, NSCLC cell line-derived xenograft (CDX) model was constructed by implanting subcutaneously modified A549 into NSG mice. Compared to conventional Msln-CAR T cells, living imaging indicated that Msln-CCR2b-CAR T cells displayed superior anti-tumor function due to enhanced migration and infiltration into tumor tissue shown by immunohistochemistry (IHC) analysis. In addition, histopathological examinations of mice organs showed that no obvious organic damages were observed. This is the first time that CAR T cell therapy combined with chemokine receptor is applied to NSCLC treatment.

## Introduction

According to 2018 global statistics of cancer incidence and mortality estimated by the International Agency for Research on Cancer, lung cancer is the most diagnosed cancer and the most common cause of cancer death (1). NSCLC accounts for ~85% of all the lung cancer, and surgery, radiotherapy, chemotherapy or targeted therapy is determined according to cancer progression. However, these treatments have some adverse effects and the 5-year survival rate is still low. In previous studies, CAR T cell therapy has attracted extensive attention, which directly recognized tumor antigen in a MHC-independent manner. CAR T cell therapy has gained remarkable success in the clinical treatment of hematological malignancies (2, 3). Three CAR T cell therapy products targeting CD19 or CD30 (Tecartus, Kymriah, and Yescarta) have been approved by Food and Drug Administration (FDA) for the treatment of hematological malignancies by the year 2020, which demonstrate the effectiveness and safety of cell products transduced with lentiviral vector (4, 5). Several clinical trials of CAR T cell therapy targeting MSLN (6–8), EGFR (9), HER2 (10, 11), CEA (12) of solid tumor have been completed, and the results were not satisfactory. CAR T cell therapy faces a number of challenges for the treatment of solid tumor, including a lack of tumor specific antigen (TSA), insufficient persistence and proliferation *in vivo*, inefficient migration and infiltration into tumor tissue, and immunosuppressive tumor microenvironment (TME) (13–15).

Mesothelin is a promising target for CAR T cell therapy (16–18), because up to 30% of cancers are detected with high levels of mesothelin expression, such as epithelial mesothelioma, pancreatic cancer, ovarian cancer, squamous cell carcinomas, adenocarcinomas and triple negative breast cancer (19–21). However, mesothelin is also expressed on normal tissue at low levels, it is a tumor-associated antigen (TAA) (22). An increased mesothelin expression was also observed in the clinical specimens and cell lines of NSCLC (23, 24). Preliminary findings from Zhao et al. showed that DNAX-activating protein 10 co-stimulation enhanced the anti-tumor efficacy of Msln-CAR T cell in NSCLC CDX and patient-derived xenograft (PDX) models (25). As of September 2020, there have been five clinical trials of Msln-CAR T cell therapy for lung cancer (NCT04489862, NCT03198052, NCT01583686, NCT02414269, and NCT03054298), four of which are still in the recruitment stage, and one terminated due to slow and insufficient accrual. Phase I study indicated that the response rate was poor and multiple side effects appeared.

One of the causes for unsatisfactory treatments for solid tumor is the limited migration of CAR T cells. Efficient migration and infiltration into tumor tissue is a precondition for potent anti-tumor activity. The trafficking process requires the interaction of chemokine secreted by tumor cell and chemokine receptor on T cells (26). Chemokines play an important role in leukocyte recruitment and are involved in angiogenesis, growth, proliferation and metastasis of tumor (27–30). Studies showed that serum levels of monocyte chemotactic protein 1 (Mcp-1) were elevated in malignant pleural mesothelioma, breast cancer and ovarian cancer patients (31–33). Cao et al. collected 50 fresh tumor tissues from NSCLC patients, and confirmed that Mcp-1 levels in tumor tissues were significantly higher than those in the tumor-adjacent and normal tissues (34). CCR2b and CCR4 are the receptors of Mcp-1 and expressed on activated T cells at low levels (35, 36). Therefore, this study generated Msln-CAR T cells modified with CCR2b or CCR4, and findings showed that Msln-CCR2b-CAR and Msln-CCR4-CAR T cells increased migration to tumor supernatants with a high level of Mcp-1 *in vitro*. Furthermore, Msln-CCR2b-CAR T cells enhanced infiltration into tumor tissue and anti-tumor function in a NSCLC CDX model.

## Materials and Methods

### Cell Lines and Primary Human Lymphocytes

NSCLC cell lines, A549 and H460, were acquired from the cell bank of National Science & Technology Infrastructure, and they were cultured in Dulbecco's modified Eagle's medium (DMEM). 293FT-17 was also maintained in DMEM. Jurkat was acquired from the American Type Culture Collection (ATCC) and cultured in RPMI 1640-media (Hyclone). All culture media were supplemented with 10% fetal bovine serum (FBS), 100 U/mL penicillin, 100 μg/mL streptomycin (Gibco). A549 and H460 cells were transduced with lentiviral vector expressing mesothelin and luciferase, and selected using 10 μg/mL puromycin for 7 days to generate A549-ML and H460-ML cell lines. Upon completion of the previous step, A549-ML and H460-ML were transduced with lentiviral vector expressing Mcp-1, and selected using 200 μg/mL hygromycin B for 14 days to generate A549-MLM and H460-MLM cell lines. All cell lines were incubated at 37°C with 5% CO_2_. Fresh blood from healthy donors were provided by Changhai Hospital, and peripheral blood mononuclear cells (PBMC) were isolated by gradient centrifugation with Ficoll-Paque Premium (GE). CD3 T cells were enriched from PBMC by negative selection with Pan T cell isolation kit (MiltenyiBiotec). According to the protocol, CD3 T cells were cultured in X-VIVO15 (Lonza) media supplemented with 5% FBS (Gibco) and stimulated by adding Dynabeads Human T-Activator CD3/CD28 (ThermoFisher Scientific) and 5 ng/mL rIL-2 (R&D system).

### Construction of Plasmids

The anti-Msln scFv was from Lanitis's article and synthesized at Genewiz (37). Msln-CAR contained CD8α signal peptide (GenBank NM001768.6, 1–63 bp), anti-Msln scFv, CD8α hinge, and transmembrane region (GenBank NM001768.6, 412–609 bp), and intracellular signaling domains of 4-1BB (GenBank NM001561.5, 640–765 bp), and CD3ζ (GenBank NM 198253.2, 154–492 bp). Msln-CAR was amplified and cloned into lentiviral backbone pTRPE. Msln-CAR and CCR2b or CCR4 were linked with 2A peptide. CCR2b and CCR4 cDNA (GenBank AAB57792.1/ABK41942.1) were provided by Prof. Jiahuai Han of Xiamen University. Mesothelin and luciferase genes were cloned into lentiviral backbone PCDH with puromycin resistance gene and Mcp-1 was overexpressed in lentiviral backbone FUGW with hygromycin resistance gene. The above plasmids were confirmed by double enzymes digestion and DNA sequencing.

### Production and Transduction of Lentiviral Vector

Lentivirus was produced based on a three-plasmid system. CAR plasmid, pSPAX2 packaging plasmid and pMD2G envelop plasmid were transfected into 293FT-17 cells in a 13.6 μg: 6.8 μg: 3.4 μg ratio using polyethylenimine (PEI) transfection regent. Lentivirus was concentrated by ultracentrifugation (Beckman Coulter). 293FT-17 cells were transduced by adding concentrated virus stock supplemented with 8 μg/mL polybrene. Lentiviral titration was performed by qPCR to determine the number of vector copies associated with genomic DNA extracted from transduced 293FT-17 cells. A prepared mix on ice was: genomic DNA template 50 ng, forward primer 1 μL, reverse primer 1 μL, 2 × SYBR GREEN master mix (Qiagen) 5 μL, and add ddH_2_O to 10 μL. Set cycling conditions as follows: 5 min at 95°C, then 40 cycles of 95°C for 10 s and 60°C for 30 s. qPCR was performed with Roche LightCycler 480 II PCR system. Plasmid was used for standard curve (WPRE-F: GGCACTGACAATTCCGTGGT, WPRE-R: AGGGACGTAGCAGAAGGACG), and human albumin gene was used as the internal control (ALB-F: GCTGTCATCTCTTGTGGGCTGT, ALB-R: ACTCATGGGAGCTGCTGGTTC). CD3 T cells were activated for 48 h, and then transduced with concentrated lentivirus in the presence of 8 μg/mL polybrene at multiplicity of infection (MOI) 15. CAR expression was detected 72 h post-transduction by flow cytometry.

### Flow Cytometry

Flow cytometry was performed on a Beckman Coulter machine, and data were analyzed using software FlowJo. Antibodies involved in this study included PE-conjugated anti-CCR2b antibody (Miltenyi Biotec), APC-conjugated anti-Msln antibody, PE-conjugated anti-CCR4 antibody (Miltenyi Biotec), FITC-conjugated recombinant mesothelin protein (ACROBiosystems), FITC-conjugated anti-CD25 antibody, PE-conjugated anti-CD69 antibody, PE-conjugated anti-HLA-DR antibody, PE-conjugated anti-CD3 antibody, FITC-conjugated anti-CD4 antibody, and PE-conjugated anti-CD8 antibody. CAR expression was detected with FITC-conjugated recombinant mesothelin protein. Other unlabeled antibodies were purchased from BD-Bioscience. Cells were stained on ice for 25 min and washed with PBS twice. Cells from peripheral blood were lysed using the red blood cell lysis buffer (Tiangen) before staining.

### Cell Proliferation Assay

To determine the effects of CCR2b and CCR4 on Msln-CAR T cell proliferation, absorbance was measured at 450 nm with a microplate reader. Resuspend 5 × 10^3^ Mock T, Msln-CAR T, Msln-CCR2b-CAR T and Msln-CCR4-CAR T cells in 100 μL culture media. Add 10 μL CCK8 (Yeasen Biotech) to each well at 12, 24, and 48 h, and incubate the 96-well plate at 37°C for 4 h.

### Transwell Assay

To confirm whether Msln-CAR T cells modified with CCR2b or CCR4 improved migration, a transwell migration assay was performed. 5 × 10^5^ Mock T, Msln-CAR T, Msln-CCR2b-CAR T and Msln-CCR4-CAR T cells were placed in the top chamber of 6.5 mm diameter, 5 μm pore size transwell (Corning). Supernatants from A549-MLM cells over 36 h were collected and placed in the bottom chamber, then the plate was incubated for 8 h at 37°C with 5% CO_2_. T cell numbers which migrated to the bottom chamber was quantified with CCK-8.

### Cytotoxicity and Cytokine Release Assay

Cytotoxicity assay was conducted based on lactate dehydrogenase (LDH) release. Target cells were A549-MLM and H460-MLM. Jurkat was used to measure the unspecific lysis. Target cells and effector cells were incubated for 8 h, and supernatants were collected. Cytotoxic activities of CAR T cells were measured at Effector/Target (E/T) ratios of 1:1, 5:1, and 10:1, respectively. Mock T cells served as control. CytoTox 96 Non-Radioactive Cytotoxicity Assay kit was purchased from Promega. CAR T cells were co-cultured with A549-MLM cells at E/T ratio of 10:1 for 24 h and supernatants were collected. The levels of IL-2, TNF-α, and IFN-γ were measured by enzyme-linked immunosorbent assay (ELISA) (Dakewe). Experiments were performed according to the manufacturer's instructions. In some experiments, to assess the safety of Msln-CCR2b-CAR T cell therapy, serum was obtained from mice orbit 28 days post-tumor cell implantation. Sample was centrifuged at 10,000 rpm, 4°C for 20 min, and supernatants were collected and used to detect the level of IFN-γ.

### NSCLC CDX Model and Anti-tumor Function of Msln-CCR2b-CAR T Cells *in vivo*

Female NSG mice aged 4–8 weeks (NOD-*Prkdc*^scid^*Il2rg*^em1^/*Smoc*, NM-NSG-001) were purchased from Shanghai Model Organisms. Mice were maintained under specific pathogen-free (SPF) conditions and provided autoclaved food and water in the animal center at Fudan University. To generate NSCLC CDX model, 5 × 10^6^ A549-MLM cells in 100 μL PBS were implanted subcutaneously into the right flanks of NSG mice on day 0, then 1 × 10^7^ CAR T cells in 100 μL PBS were injected via the tail vein on day 7. Effector cells were injected for the second time on day 14, and PBS was injected as control. Bioluminescence imaging was conducted once a week to monitor the tumor change. Mice were anesthetized by injecting intraperitoneally pentobarbital sodium. Each mouse was injected 150 mg luciferin/kg body weight via tail vein 5 min before living imaging. Set exposure time to 2 min, the upper and lower thresholds of bioluminescence intensity to 65,535 and 6,500 cps, respectively. Living imaging was performed with bioanalytical instruments purchased from Bathold Technologies and data were acquired with software IndiGO.

### Quantitative Real-Time PCR

Genomic DNA were extracted from PBMC of CAR T cells-treated mice. According to the instructions of QuantiNova SYBR Green PCR Kit, all reactions were conducted on a Roche LightCycler 480 II PCR machine with the abovementioned primers WPRE-F and WPRE-R. Human GAPDH (GAPDH-F: TCAAGTGGGGCGATGCTGGC, GAPDH-R: TGGGGGCATCAGCAGAGGGG) served as the internal control. qPCR was used to assess the persistence of Msln-CAR and Msln-CCR2b-CAR T cells in the peripheral blood.

### Histopathological Examination

Mice with tumor that reached 15 mm in length were sacrificed and the heart, liver, spleen, lung, kidney, and tumor masses were dissected. Tissue specimens were fixed with 4% buffered formaldehyde. Paraffin sections were used for hematoxylin and eosin (H&E) staining and IHC analysis. H&E stainings of major organs were used for the preliminary safety evaluation of CAR T cell *in vivo*, and IHC stainings of spleen and tumor were used to assess the persistence and infiltration of CAR T cells. The primary and secondary antibodies were rabbit anti-human CD3 antibody and HRP-conjugated goat anti-rabbit IgG H&L (Abcam), respectively. Nuclei were then stained with DAPI (Abcam). Tumors of PBS-treated mice were stained as control. In order to determine if CCR2b and CCR4 induced Msln-CAR T cell to migrate more effectively, positive CD3 T cells in IHC section were enumerated with multi-point cell counter of Image J.

### Statistical Analysis

GraphPad Prism was used for statistical analysis, and data were shown as the means ± SEM. Student's unpaired *t*-test was used to compare statistical difference between groups, and one-way ANOVA or two-way ANOVA was used for comparison among multiple groups. ^*^P < 0.05 was considered to be significant.

## Results

### Construction and Expression of CAR

**Figure 1:**
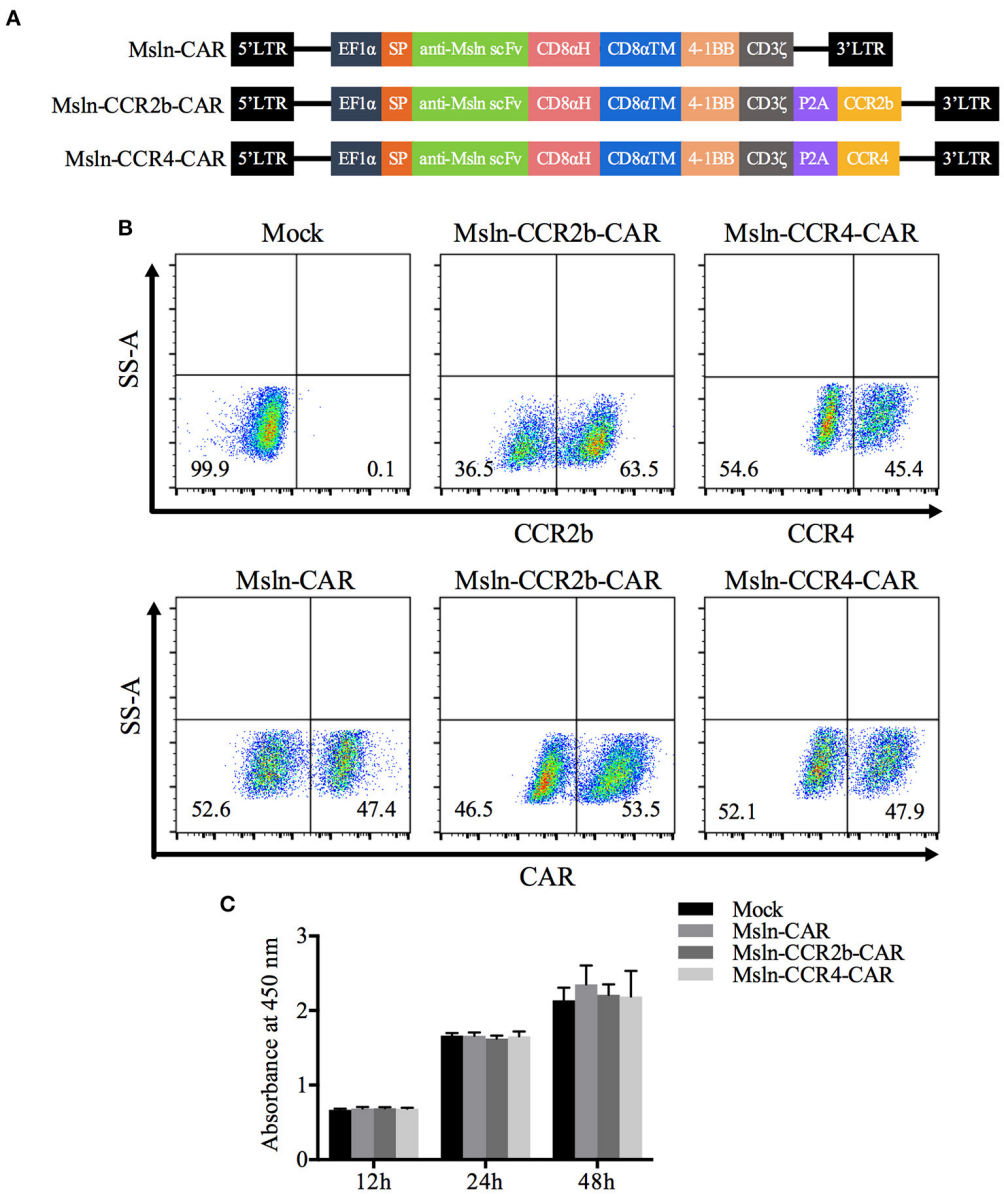
Construction and expression of CAR in primary CD3 T cells. **(A)** Structural diagram of Msln-CAR alone or in combination with CCR2b or CCR4. The CAR consisted of signal peptide, anti-Msln scFv, CD8α hinge and transmembrane domain, 4-1BB co-stimulatory domain, and CD3ζ cytoplasmic domain. LTR, long terminal repeat; EF1α, promoter; SP, signal peptide; scFv, single-chain variable fragment; CD8αH, CD8α hinge; TM, transmembrane; CCR, cell chemokine receptor. **(B)** CD3 T cells were transduced with lentiviral vector expressing Msln-CAR, Msln-CCR2b-CAR, or Msln-CCR4-CAR. Expressions of CCR2b and CCR4 were confirmed by staining with PE-conjugated anti-CCR2b antibody and PE-conjugated anti-CCR4 antibody, respectively. CAR expression was evaluated by staining with FITC-conjugated recombinant mesothelin protein 72 h post-transduction, and CD3 T cells transduced with empty lentiviral vector were stained as control. **(C)** Proliferation ability of Msln-CAR, Msln-CCR2b-CAR, and Msln-CCR4-CAR T cells at different time points. Experiments were repeated for three times, and data represented the means ± SEM. *P*-value was calculated by two-way ANOVA.

To obtain Msln-CCR2b-CAR and Msln-CCR4-CAR T cells, we generated a tandem lentiviral vector encoding the Msln-CAR and CCR2b or CCR4 with P2A peptide sequence between genes. CAR contained CD8α signal peptide, anti-Msln scFv (37), CD8α hinge and transmembrane domain, 4-1BB co-stimulatory domain, and CD3ζ signaling domain (Figure 1A). PBMC from healthy donor were isolated by gradient centrifugation, and CD3 T cells were enriched by negative selection. FACS analysis showed that the population of enriched cells contained 89% of CD3 T cells, and the percentage of CD4 and CD8 T cell subpopulations was 55 vs. 33. Next, CD3 T cells were activated by using CD3/CD28 dynabeads for 48 h, with expressions of CD25, CD69, and HLA-DR tested. Results showed that 75% of CD3 T cells were CD25-positive, and 83% CD69-positive. FACS analysis indicated that CD3 T cells were in the early to middle stage of activation (results not shown). Primary CD3 T cells were transduced with lentiviral vectors expressing Msln-CAR, Msln-CCR2b-CAR, or Msln-CCR4-CAR. Expressions of CAR were tested by FACS 72 h post-transduction. Results showed that transduction efficiencies reached 47% (Msln-CAR), 54% (Msln-CCR2b-CAR), and 48% (Msln-CCR4-CAR), respectively, and there were no significant differences among three groups. Expression efficiencies of CCR2b and CCR4 were 64 and 45%, while primary CD3 T cells transduced with empty lentiviral vector didn't express CAR and CCR2b or CCR4 (Figure 1B). Compared to Mock T cells, CCR2b or CCR4 modification didn't have an effect on proliferation activity of Msln-CAR T cells by CCK-8 test (Figure 1C).

### Generation of NSCLC Cell Lines Expressing Mesothelin, Luciferase, and Mcp-1

**Figure 2:**
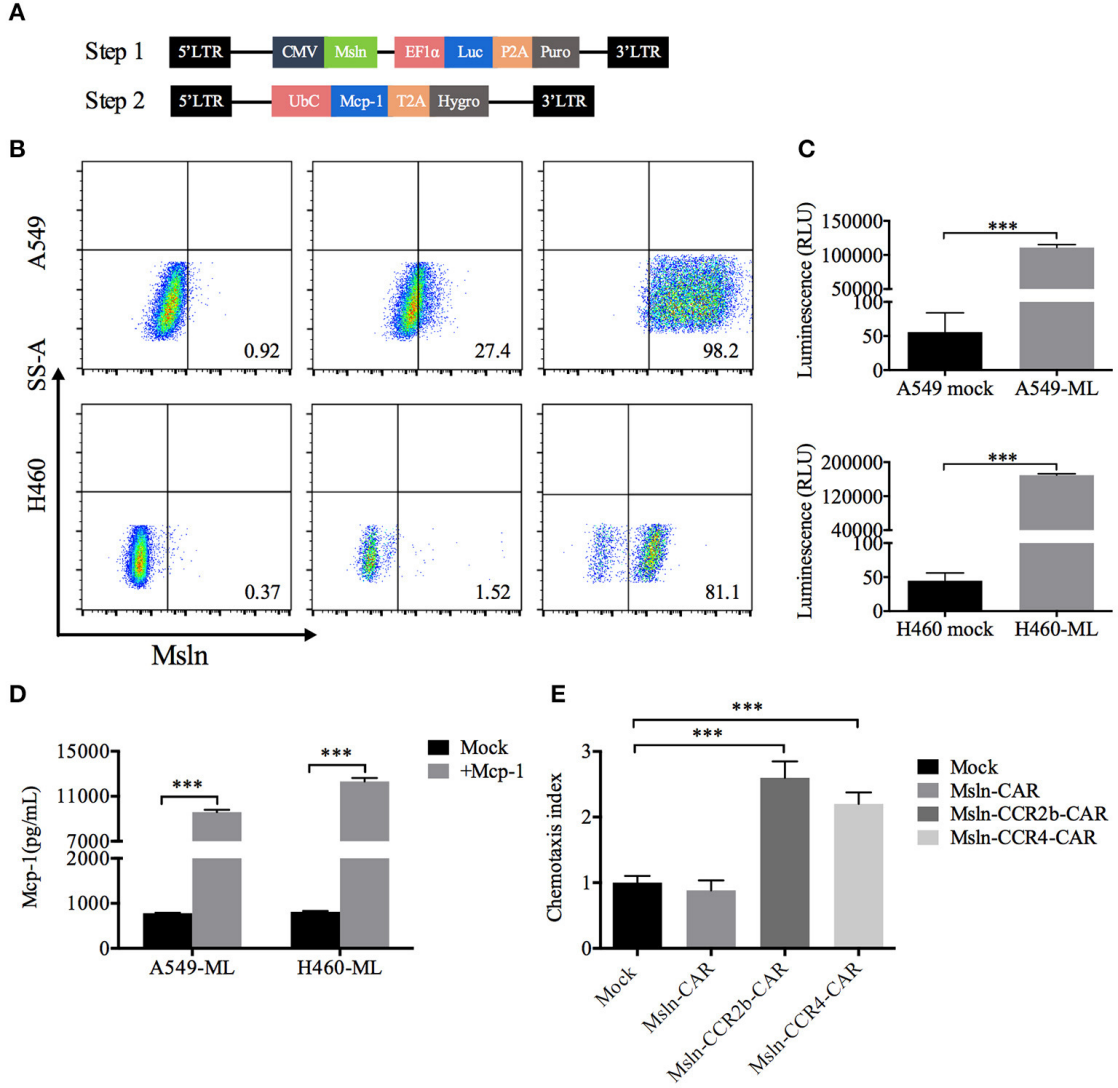
Generation of target cells and functional assays of CCR2b and CCR4 *in vitro*. **(A)** Schematic diagram of lentiviral vector (Step 1) encoding mesothelin and luciferase. LTR, long terminal repeat; CMV, promoter; Msln, mesothelin; EF1α, promoter; luc, luciferase; puro, puromycin resistance gene. Schematic diagram of lentiviral vector (Step 2) encoding Mcp-1. UbC, promoter; Mcp-1, monocyte chemotactic protein 1; Hygro, hygromycin resistance gene. **(B)** Surface mesothelin expressions of human NSCLC cell lines A549 and H460 were detected by FACS. The native A549 and H460 cells were engineered to express high levels of mesothelin and luciferase with lentiviral vector (Step 1). **(C)** Luciferase expression was quantified by measuring luminescence using a multimode plate-reader. Data represented the means ± SEM of triplicate wells. Results were representative of three independent repeats. *P*-values were calculated by Student's *t*-test. ***P < 0.001. **(D)** A549-ML and H460-ML cells were transduced with the abovementioned vector (Step 2) and selected in hygromycin for 14 days (A549-MLM and H460-MLM). Supernatants from A549-MLM and H460-MLM cells were collected, and Mcp-1 expression was quantified by ELISA. Supernatants from native A549-ML and H460-ML cells served as the blank control. Each experiment was set up in triplicate wells, and results from three independent replications represented the means ± SEM. ***P < 0.001. *P*-values were calculated by Student's *t*-test. **(E)** A549-MLM cell supernatants induced Msln-CCR2b-CAR and Msln-CCR4-CAR T cells migration in a Mcp-1-dependent manner. One hundred microliters CAR T cells were placed in the top chamber, and 600 μL supernatants of A549-MLM cells were placed in the bottom chamber. Total T cell number that migrated to the bottom chamber was quantified by CCK-8. The test was replicated for three times and each experiment was set up in triplicate wells. Data represented the means ± SEM. *P*-values were calculated by one-way ANOVA. ***P < 0.001.

Taking NSCLC cell lines A549 and H460 as the research objects, target cells were generated in two steps. Firstly, A549 and H460 cells were transduced with lentiviral vector encoding mesothelin and luciferase, and selected with puromycin for 7 days to generate the target cells A549-ML and H460-ML. A549-MLM and H460-MLM cells were generated by transducing A549-ML and H460-ML with a lentiviral vector expressing Mcp-1 and hygromycin resistance gene upon completion of the first step (Figure 2A). FACS analysis indicated that expression efficiencies of mesothelin increased to 98 and 81% relative to parental cell lines, respectively. Luciferase was used for living imaging (Figures 2B,C). ELISA analysis indicated Mcp-1 concentrations from A549-ML and H460-ML cell supernatants were <1,000 pg/mL/10^6^ cells. After selections were completed, Mcp-1 concentrations from A549-MLM and H460-MLM cell supernatants reached 9,596 and 12,319 pg/mL/10^6^ cells, respectively (Figure 2D).

### Effect of CCR2b and CCR4 on Msln-CAR T Cells Migration *in vitro*

The functional activity of CCR2b and CCR4 was tested *in vitro*. Transwell assay revealed that T cells could migrate through 5 μm polycarbonate membrane to the bottom chamber containing cell supernatants from A549-MLM cells. Relative to Mock T cells, migration efficiency of Msln-CCR2b-CAR and Msln-CCR4-CAR T cells increased to 2.6/2.2 times, respectively, and there was no difference for conventional Msln-CAR T cells (Figure 2E). Therefore, CCR2b and CCR4 were functional in response to Mcp-1 secreted by tumor cells *in vitro*.

### Specific Cytotoxicity of Msln-CCR2b-CAR and Msln-CCR4-CAR T Cells

**Figure 3:**
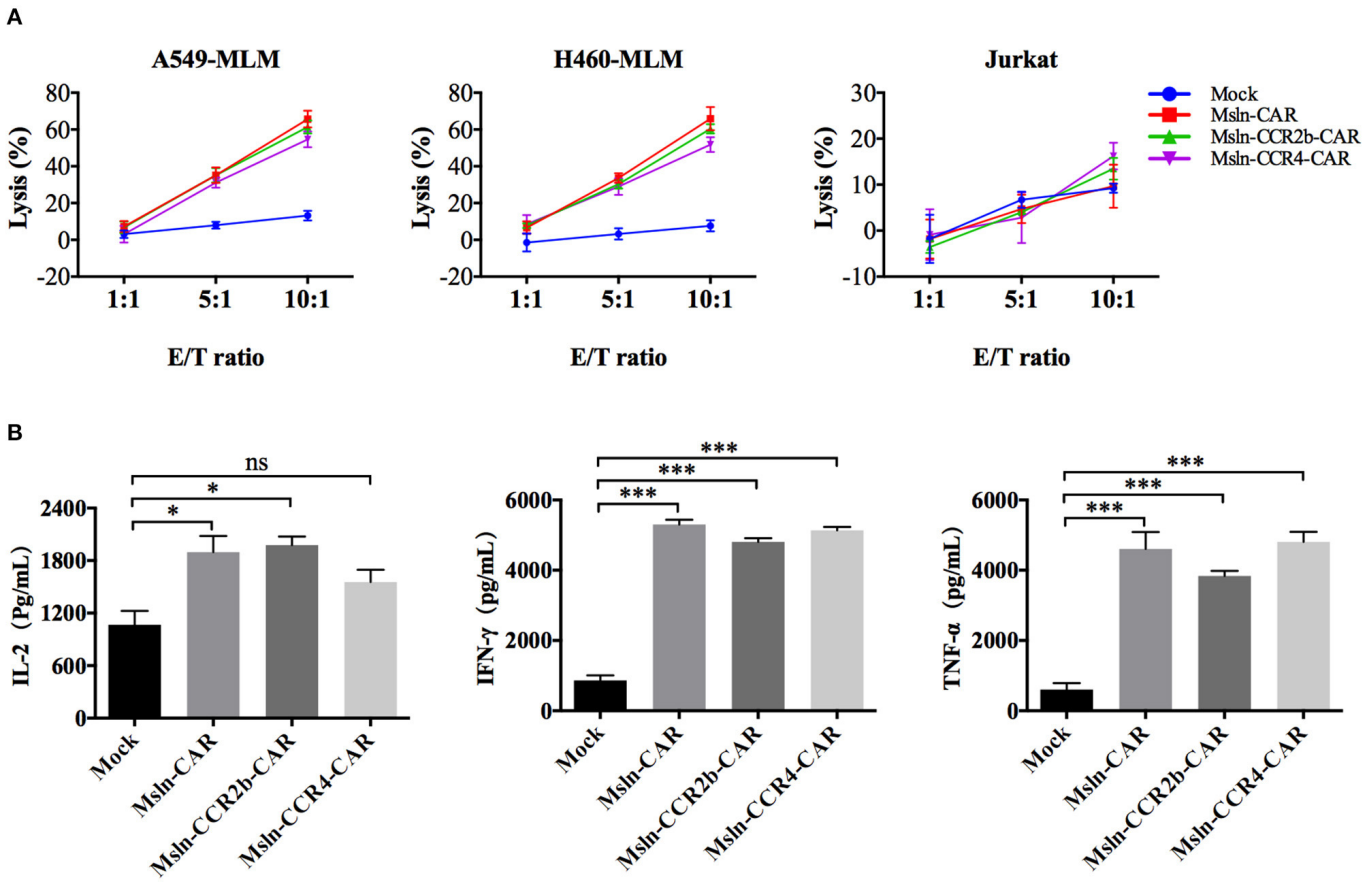
Msln-CCR2b-CAR T cells specifically killed mesothelin-positive tumor cells and increased cytokine release. **(A)** Target cells (A549-MLM, H460-MLM, and Jurkat cells) were co-incubated with different effector cells at the indicated E/T ratios for 8 h. Cytotoxicity was quantified based on LDH release. Data represented the means ± SEM. Experiments were repeated for three times, and *P*-values were calculated using two-way ANOVA. **(B)** CAR T cells were co-incubated with A549-MLM for 24 h and supernatants were collected for ELISA. Cytokines secreted by CAR T cells were quantified from three independent repeats. Data represented the means ± SEM, and one-way ANOVA was used for statistical analysis. ns, not significant. *P < 0.05 and ***P < 0.001.

After co-incubation with mesothelin-positive target cells for 8 h, Msln-CAR, Msln-CCR2b-CAR, and Msln-CCR4-CAR T cells specifically lysed A549-MLM and H460-MLM cells equivalently at different E/T ratios, while neither lysed mesothelin-negative Jurkat cells based on LDH release. The cytolytic efficiencies of Msln-CCR2b-CAR and Msln-CCR4-CAR T cells reached 52–66% at 10:1 E/T ratio, and cytotoxicity was positively correlated with the E/T ratio. CCR2b or CCR4 modification didn't increase cytolytic activity of Msln-CAR T cells *in vitro* (Figure 3A). Compared to Msln-CCR4-CAR, cytolytic activity of Msln-CCR2b-CAR T cells was slightly higher after co-incubation with target cells. After co-incubation with A549-MLM cells at 10:1 E/T ratio for 24 h, Msln-CAR, Msln-CCR2b-CAR, and Msln-CCR4-CAR T cells produced high levels of IL-2, IFN-γ, and TNF-α compared to Mock T cells, but cytokine levels among three CAR T cell groups were similar (Figure 3B). Mesothelin expression of A549-MLM was higher relative to that of H460-MLM. For this reason, A549-MLM was chosen as the research object for further cytokine analysis and anti-tumor assay *in vivo*.

### Anti-tumor Activity of Msln-CCR2b-CAR T Cells in a NSCLC CDX Model

**Figure 4:**
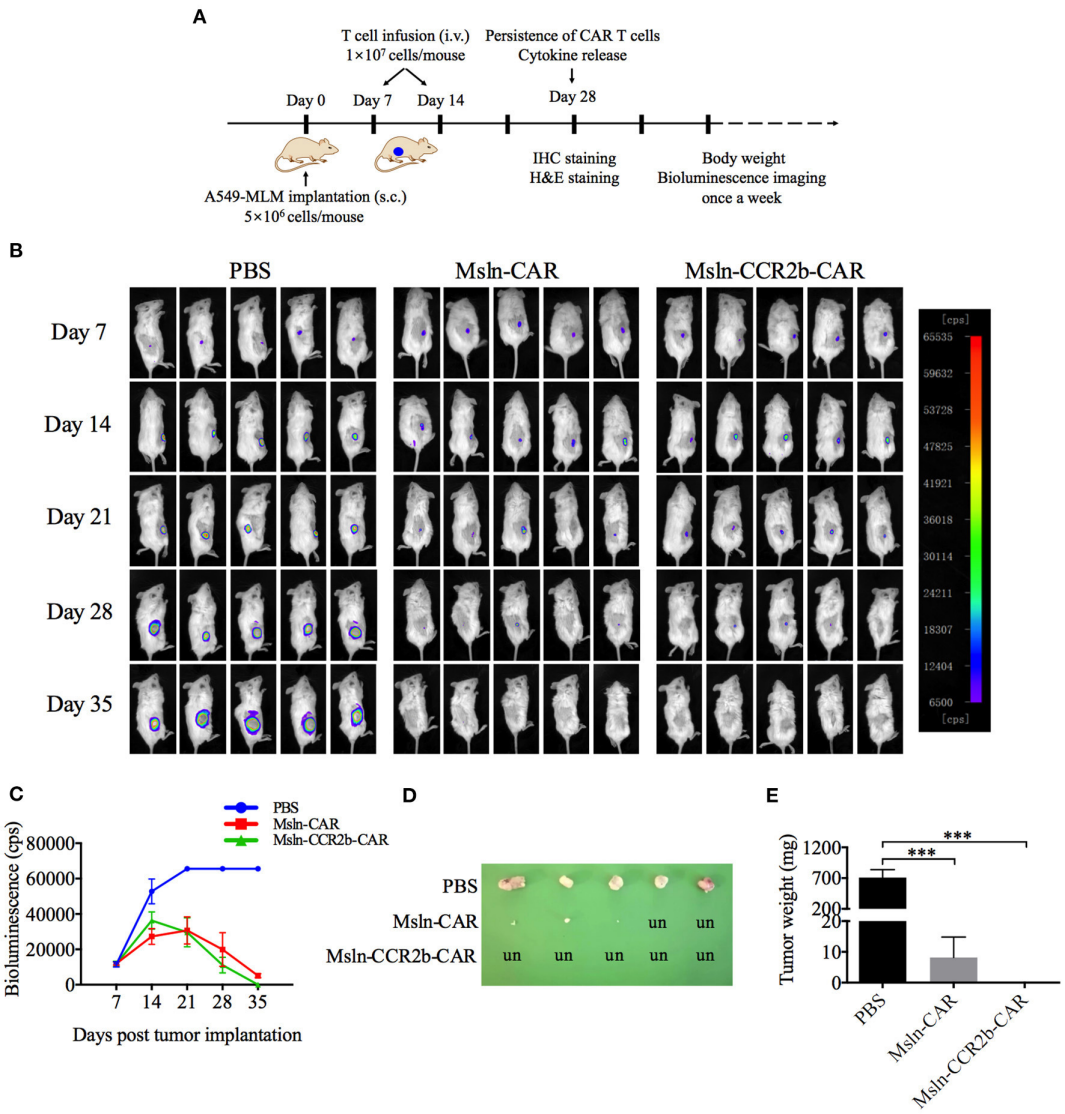
CCR2b enhanced anti-tumor function of Msln-CAR T cells *in vivo*. **(A)** Schematic diagram of experiments *in vivo*. A549-MLM cells were implanted subcutaneously into 6-week-old female NSG mice. Seven days post-tumor cell implantation, mice were infused intravenously with PBS, Msln-CAR T or Msln-CCR2b-CAR T cells (1 × 10^7^ cells per mouse). The second injection of effector cells was performed 7 days later. Tumor growth was monitored by bioluminescence imaging every 7 days. s.c., subcutaneous; i.v., intravenous. **(B)** Sequential bioluminescence imaging of mice injected with PBS, Msln-CAR T, or Msln-CCR2b-CAR T cells were shown (*n* = 5 mice per group in three independent experiments). **(C)** Bioluminescence intensity of tumor cells at different time points was captured. Data represented the means ± SEM for 5 mice per group. *P*-values were calculated by two-way ANOVA. ***P < 0.001. **(D,E)** Tumor size and weight of different groups. un, undetectable. Tumor weights were analyzed by one-way ANOVA, and data represented the means ± SEM. ***P < 0.001.

After confirming the function of Msln-CCR2b-CAR T cells *in vitro*, a NSCLC CDX model was constructed by implanting subcutaneously A549-MLM cell into the right flanks of NSG mice (Figure 4A). The first living imaging was conducted 7 days post-tumor cell transplantation. Figure 4B showed that obvious bioluminescence (10,000 cps) was detected in all 15 mice, and this indicated successful implantation of tumor cells. We detected that more than 80% of effector cells were in naïve (CD45RA^+^ and CD62L^+^), central memory (CD45RA^−^ and CD62L^+^), and effector memory (CD45RA^−^ and CD62L^−^) phenotypes 10 days post-transduction. Then, CAR T cells or PBS were injected via tail vein. Tumor continued to grow as time went on in PBS-treated group and Msln-CAR T and Msln-CCR2b-CAR T cells slowed down tumor growth visibly compared to PBS. Living imaging indicated that tumors were completely eliminated in 40% of Msln-CAR-treated mice and 100% of Msln-CCR2b-CAR-treated mice 35 days post-tumor implantation. Meanwhile, bioluminescence intensity at different time points were quantified, and results showed that bioluminescence value of Msln-CCR2b-CAR group was much lower than Msln-CAR group, indicating a better anti-tumor function of Msln-CCR2b-CAR T cells (Figure 4C). Furthermore, tumors from all mice were dissected and tumor weight was quantified. We found that tumor growth was inhibited significantly in Msln-CAR and Msln-CCR2b-CAR-treated mice, and tumor was completely eliminated in Msln-CCR2b-CAR-treated mice (Figures 4D,E), consistent with results of living imaging analysis.

### Persistence of Msln-CCR2b-CAR T Cells in the Peripheral Blood, Major Organs, and Tumor

**Figure 5:**
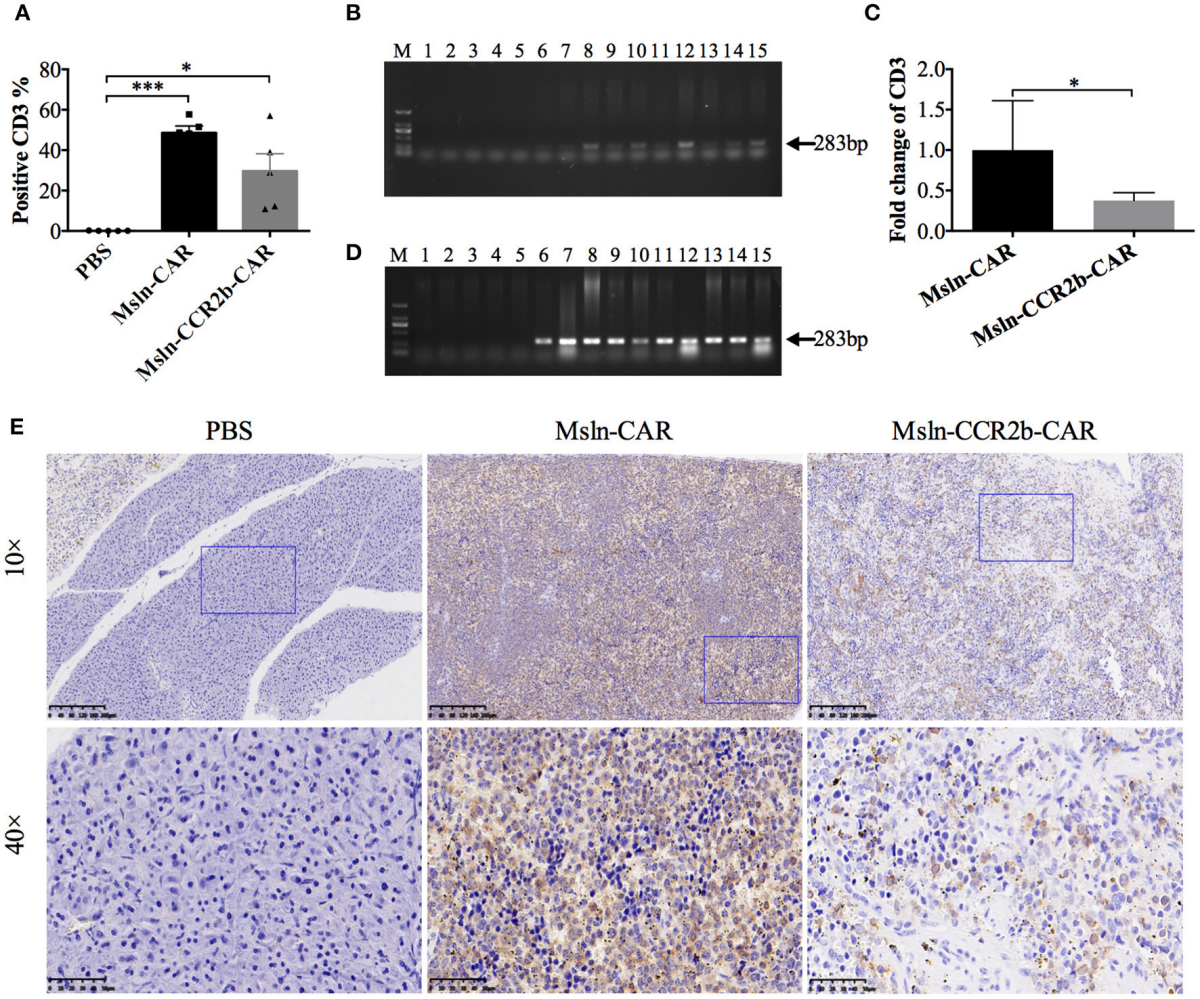
Persistence and proliferation of CAR T cells *in vivo*. **(A)** Peripheral blood analysis of the proportion of CAR T cells 28 days post-tumor implantation by FACS. One hundred microliters peripheral blood was collected from mouse orbit, and proportion of CAR T cells was detected with PE-conjugated anti-CD3 antibody after the red blood cell lysis. Statistical differences were analyzed by one-way ANOVA, and data represented the means ± SEM. Experiments were repeated for three times. *P < 0.05 and ***P < 0.001. **(B)** Peripheral blood analysis of persistence of CAR T cells 28 days post-tumor cells implantation by PCR. One hundred microliters peripheral blood was collected from mouse orbit, and genomic DNA were extracted after the red blood cells lysis. PCR was performed with the specific primers designed for anti-Msln scFv. The expected positions (283 bp) of the resulting DNA bands were indicated by an arrow at the right of the gel. M, DNA marker; lanes 1–5, amplicons for representative 5 mice injected with PBS; lanes 6–10, amplicons for representative 5 mice injected with Msln-CAR T cells; lanes 11–15, amplicons for representative 5 mice injected with Msln-CCR2b CAR T cells. **(C)** Genomic DNA from PBMC of Msln-CAR and Msln-CCR2b-CAR-treated mice were subjected to qPCR with specific primers for anti-Msln scFv and GAPDH. Experiments were repeated for three times. Error bar represented ± SEM and *P*-value was calculated by Student's *t*-test. *P < 0.05. **(D)** Organic analysis of persistence of CAR T cells 28 days post-tumor cells implantation by PCR. Major organs (heart, liver, spleen, lung, and kidney) were dissected from mice treated with PBS, Msln-CAR, or Msln-CCR2b-CAR T cells and genomic DNA were extracted. PCR was performed with specific primers designed for anti-Msln scFv. Expected positions (283 bp) of the resulting DNA bands were indicated by an arrow at the right of the gel. M, DNA marker; Lanes 1–15 represent amplicons for heart, liver, spleen, lung, and kidney of representative 3 mice injected with PBS (lanes 1–5), Msln-CAR T (lanes 6–10), or Msln-CCR2b-CAR T cells (lanes 11–15), respectively. **(E)** Spleens of PBS, Msln-CAR, or Msln-CCR2b-CAR-treated mice were stained with specific rabbit anti-human CD3 mAb and HRP-conjugated goat anti-rabbit IgG H&L. The rectangular area in the 10× picture was enlarged to 40×. Scale bars: 200 μm for 10×, 50 μm for 40×.

We detected the persistence and proliferation of Msln-CAR T and Msln-CCR2b-CAR T cells in the peripheral blood of each mouse 28 days post-tumor cell implantation (Supplementary Figure 1). FACS analysis showed that average percentage of CD3 T cells was 49% in Msln-CAR-treated mice, higher than Msln-CCR2b-CAR-treated mice (30%) (Figure 5A). Meanwhile, genomic DNA of cells in the peripheral blood were extracted and amplified with specific primers designed for anti-Msln scFv. PCR results indicated that expected 283 bp DNA bands were only detected in Msln-CAR and Msln-CCR2b-CAR-treated groups (Figure 5B). Furthermore, in order to compare the persistence of Msln-CAR and Msln-CCR2b-CAR T cells in the peripheral blood, genomic DNA were subjected to qPCR with above primers. qPCR analysis indicated that the number of Msln-CAR T cells was 2.7 times of Msln-CCR2b-CAR T cells (Figure 5C). At the same time, human IFN-γ secretion in the peripheral blood was detected. ELISA analysis showed that IFN-γ concentrations were 576 and 662 pg/mL 21 days post-injection of Msln-CAR or Msln-CCR2b-CAR T cells, respectively (Supplementary Figure 2). In addition, heart, liver, spleen, lung and kidney of mice were dissected. Genomic DNA of abovementioned organs were extracted and amplified with primers specific for anti-Msln scFv, and PCR results showed that Msln-CAR and Msln-CCR2b-CAR T cells could migrate and infiltrate into organs (Figure 5D). Furthermore, spleen was selected as representative organ for detailed analysis. IHC staining revealed that CD3-positive T cells were detected in Msln-CAR and Msln-CCR2b-CAR-treated mice (Figure 5E). Meanwhile, H&E staining of tumor tissue showed that only A549-MLM cells were observed in PBS-treated mice, and cells were fibrous with relatively large nuclei. In Msln-CAR and Msln-CCR2b-CAR groups, a number of lymphocytes were distributed around tumor cells. Furthermore, this study showed that A549-MLM cells were apoptotic around the lymphocytes, indicating that partial tumor cells had been lysed by CAR T cells (Supplementary Figure 3).

### Increased Migration and Infiltration of Msln-CCR2b-CAR T Cells *in vivo*

**Figure 6:**
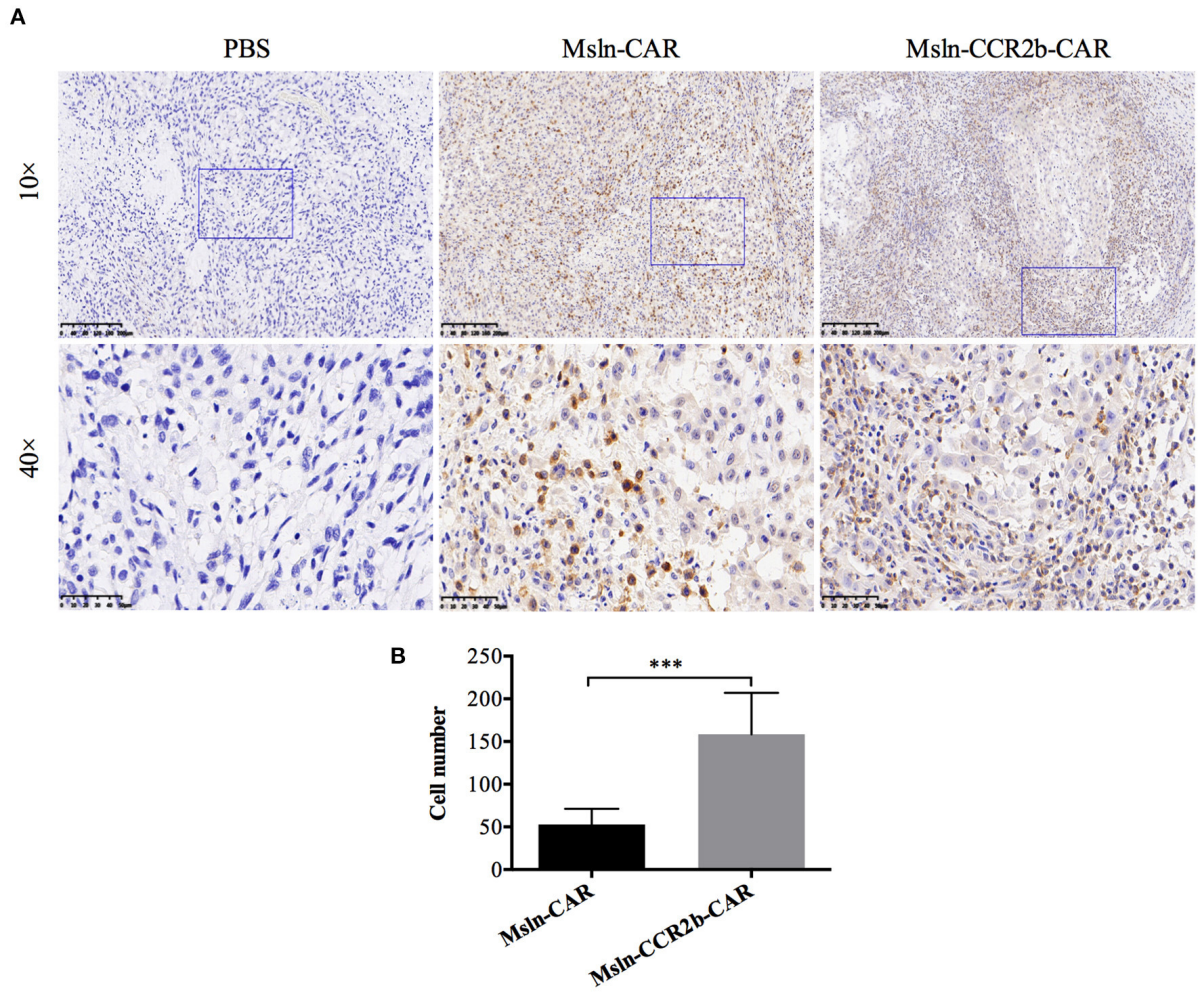
CCR2b increased migration and infiltration of Msln-CAR T cells into tumor tissue. **(A)** Paraffin sections of tumors from three groups were stained with specific rabbit anti-CD3 mAb and HRP-conjugated goat anti-rabbit IgG H&L. CD3 T cells were detected in the tumor region of Msln-CAR group and Msln-CCR2b-CAR group whereas no CD3 T cells were observed in the tumor mass of PBS group. The rectangular area in the 10× picture was enlarged to 40×. Scale bars: 200 μm for 10×, 50 μm for 40×. **(B)** Four fields of tumor sections were randomly selected, and positive CD3 T cells (yellow) were enumerated manually using the multi-point cell counter of Image J. Data were representative of the means ± SEM. *P*-value and statistical differences were analyzed by Student's *t*-test. ***P < 0.001.

To determine whether CCR2b improved Msln-CAR T cells migration *in vivo*, we preformed IHC staining of tumors 28 days post-tumor implantation. Results indicated that massive CD3-positive T cells infiltrated into the tumor tissue in CAR T cells-treated mice (Figure 6A). Furthermore, number of CD3 T cells in IHC section was manually enumerated with the multi-point cell counter. We found that number of CD3 T cells of Msln-CCR2b-CAR-treated mice was 3 times of Msln-CAR-treated mice (Figure 6B). Compared to the conventional Msln-CAR T cells, CCR2b modification enhanced the migration and infiltration of Msln-CAR T cells into tumor tissue.

## Discussion

Now more and more studies focus on the treatment of solid tumors, including lung cancer, the largest cancer patient group. The mortality rate of lung cancer is high under traditional treatments. According to the ClinicalTrials.gov., most clinical trials of CAR T cell immunotherapy for lung cancer recruit patients with terminal cancer, which has recurred or progressed after multi-mode therapies. Therefore, CAR T cell therapy is a potential treatment for lung cancer. Studies from different teams have indicated that CAR T cell functions were improved by co-expressing responding CCR. The first report on CAR T cells co-expressing CCR was from Di Stasi's team where they revealed that CCR4-modified CD30-CAR T cells improved homing and anti-tumor activity in a Hodgkin tumor model (38). Similarly, Craddock et al. demonstrated that CCR2b-modified GD2-CAR enhanced tumor trafficking in a neuroblastoma model (39), and Moon et al. revealed that CCR2-modified Msln-CAR T cells enhanced tumor eradication in a pleural mesotheliomas model (40). Moreover, they detected that Msln-CAR T cells were able to kill more tumor cells in *vitro* by modifying with CCR2b. However, this phenomenon was not observed in this study. Compared to Msln-CAR T cells, proliferation activity and cytotoxicity of Msln-CCR2b-CAR T cells did not increase *in vitro*, and this was consistent with Jin's study (41). Anti-tumor function of Msln-CCR2b-CAR T cells was detected by living imaging because transplanted tumor cells were luciferase-labeled. We think that enhanced anti-tumor activity means increased migration and infiltration of Msln-CCR2b-CAR T cells. The limitation is that bioluminescence imaging of tumor cells is an indirect method to monitor T cell location. In order to directly monitor CD70-CAR T cell modified with CXCR-1 or CXCR-2 *in vivo*, Jin designed a double expression system. The near-infrared protein iRFP720 was overexpressed in tumor cells, and click beetle luciferase (CBluc) was overexpressed in T cells. Therefore, co-localization of T cells and tumor cells were able to be detected by living imaging, and effects of CXCR-1 and CXCR-2 on T cell trafficking were observed directly (41). Unlike the previous studies, data of living imaging were supported by further IHC analysis, and more CD3-positive T cells were detected in Msln-CCR2b-CAR-treated tumor. Furthermore, FACS analysis demonstrated that there were much less circulating CD3 T cells in peripheral blood of Msln-CCR2b-CAR-treated mice and the results were consistent with the previous study (40). In order to compare the difference of anti-tumor activity *in vivo*, serial bioluminescence intensity was quantified. Generally speaking, total photon emission of tumor cells need to be measured. However, in this study, our team analyzed the maximum bioluminescence value. The reason was that the maximum threshold of the instrument we used was 65,535 cps. Photo emission of tumor cells in PBS-treated mice reached the upper limit 21 days post-tumor cells implantation and entered the plateau stage as shown in Figure 4C. Due to the limitation of the instrument, we were not able to calculate the total photo emission.

As shown in living imaging, there was no significant difference between Msln-CAR and Msln-CCR2b-CAR. The main reason was that CAR T cells were injected at an earlier time point (7 days post-tumor transplantation), and tumor volume was not large enough at that time. Generally, CAR T cells will be injected when tumor volume reaches 100–200 mm^3^. But the living imaging system we used has a low sensitivity. If tumor volume is too large, fluorescence intensity will exceed the maximum detectable threshold. In order to observe the difference between Msln-CAR and Msln-CCR2b-CAR more clearly, we will postpone the time point of CAR T cells injection or reduce the dose of injection in the future study.

Body weight of mice were recorded at sequential time points. Results showed that no body weight loss was observed for all mice after the first injection of effector cells, and there was a small decrease after the second injection of Msln-CAR T or Msln-CCR2b-CAR T cells compared to mice treated with PBS (Supplementary Figure 4). A slight GvHD was produced due to the infusion of 2 × 10^7^ CAR T cells (42). In addition, histopathological examinations for heart, liver, spleen, lung, and kidney showed that no obvious tissue damages were observed (Supplementary Figure 5). An important indicator of clinical evaluation of CAR T cell therapy is the prolonged survival of patients. For the preclinical study, there was a need to prolong the survival of tumor-bearing mice. In this study, the survival curve of mice was not shown since tumor growth was inhibited, even with the eliminated after injection of CAR T cells over time. On the contrary, tumors of PBS-treated mice continued growth with a diameter of more than 15 mm. Worsely, some tumors were already ulcerated and necrotic due to the overlarge size. Even so, progressing tumors did not result in the death of PBS-treated mice. According to the ethical requirement of small animal experiments, mice were sacrificed by cervical dislocation. Therefore, the survival curve was not able to reflect the true survival time in this study. NSG mouse model are used to evaluate and optimize Msln-CCR2b-CAR in this study, and safety evaluation associated with CAR T cells can't be observed in this model, especially for cytokine storm. Host immune system including immune and non-immune cells are essential for toxicity evaluation. Unlike cell lines, patient-derived tumor or normal tissues are heterogeneous and contain a variety of cell types. Toxicity can be better studied based on further modified NSG mouse models (NSG-SGM3, NSG-MHC-DKO, and so on), PDX models or immune intact mouse models using mouse CAR T cells.

As mentioned above, Mcp-1 is overexpressed in serum and tumor tissue of patients and Mcp-1 level is associated with overall survival and progression-free survival. As a third-line treatment for NSCLC, anlotinib was reported a novel anti-angiogenetic mechanism via inhibiting Mcp-1 (30, 43). Future studies may call for Mcp-1 to be used as a biomarker to monitor and predict the clinical outcomes of some treatments. This study was the first report about Msln-CAR T cell therapy modified with CCR2b for the treatment of NSCLC. We directly observed increased migration and infiltration into tumor tissue by IHC analysis rather than FACS analysis in Msln-CCR2b-CAR-treated mice. Except for CCR2b, our team will conduct further studies on other chemokine receptors, such as CCR6 and CXCR5. The corresponding chemokines (CCL20 and CXCL13) are high-expressed in lung adenocarcinoma and squamous cell carcinoma. What's more, CAR T cells modified with CCR6 or CXCL5 have not been reported. As a preclinical trial, this study will provide the foundation for our future work, and we hope that more optimized CAR T cells can be applied to clinical trials as soon as possible.

## Data Availability Statement

The original contributions presented in the study are included in the article/Supplementary Material, further inquiries can be directed to the corresponding author/s.

## Ethics Statement

The animal study was reviewed and approved by Animal Ethic Committee of School of Life Sciences at Fudan University and Medical Ethics Committee of Changhai Hospital.

## Author Contributions

HZ conceived and designed the experiment. Functional assay of Msln-CCR2b-CAR T cells *in vitro* was conducted by YW. Animal experiments were performed with the help of JW, JY, and PL. YW wrote the manuscript and analyzed the data. Language editing was provided by BL. All authors discussed the results, revised the manuscript, and approved the submission.

## Conflict of Interest

The authors declare that the research was conducted in the absence of any commercial or financial relationships that could be construed as a potential conflict of interest.
