# Supplementary material for: Chemokine Receptor CCR2b Enhanced Anti-tumor Function of Chimeric Antigen Receptor T Cells Targeting Mesothelin in a Non-small-cell Lung Carcinoma Model
Source: Front Immunol. 2021 Mar 11;12:628906. doi: 10.3389/fimmu.2021.628906 (PMC7992009; doi:10.3389/fimmu.2021.628906)
Supplement: Supplementary file 1 [file Data_Sheet_1.PDF]

frontiers

Supplementary Material

Supplementary Figures

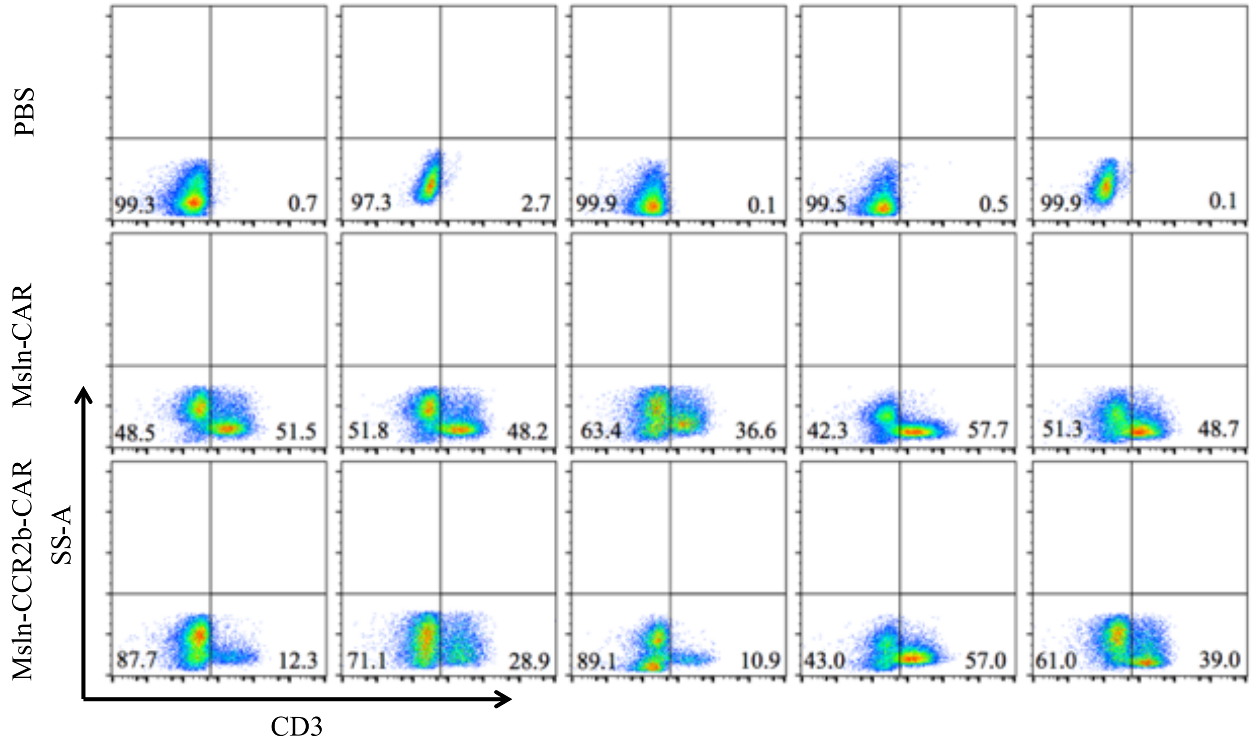

Supplementary Figure 1. Persistence and proliferation of CAR T cells *in vivo.* Peripheral blood analysis of the proportion of CAR T cells 28 days post tumor implantation by FACS. 100 µL peripheral blood was collected from mouse orbit, and proportion of CAR T cells was detected with PE-conjugated anti-CD3 antibody for all 15 mice.

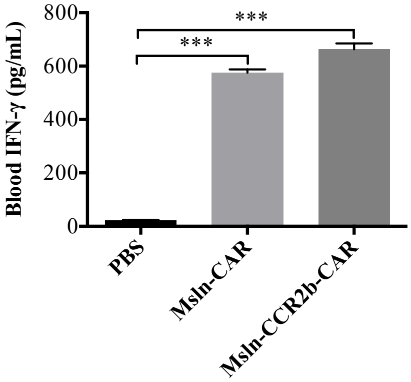

Supplementary Figure 2. IFN-γ level in the peripheral blood was detected by ELISA. Results represented the means ± SEM and one-way ANOVA was used for P values and statistical differences analysis. P<0.05(*), P<0.01(**), P<0.001(***).

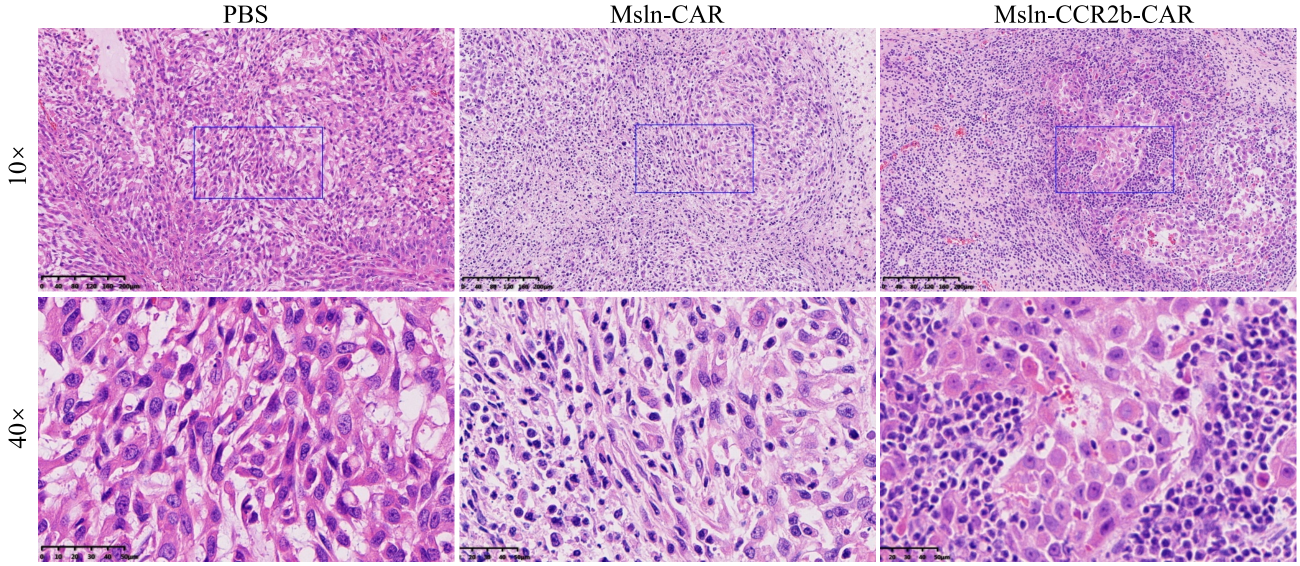

Supplementary Figure 3. Persistence and proliferation of CAR T cells in tumor. Representative H&E staining of tumor tissues. The rectangular area in the 10× picture was enlarged to 40×. Scale bars: 200 μm for 10×.

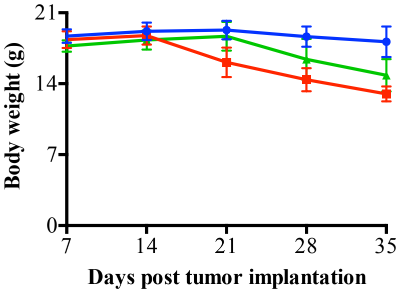

Supplementary Figure 4. Sequential body weight was analyzed every 7 days after tumor cells implantation. Data represented the means ± SEM in 3 independent repeats and P value was calculated using two-way ANOVA. P<0.05(*), P<0.01(**), P<0.001(***).

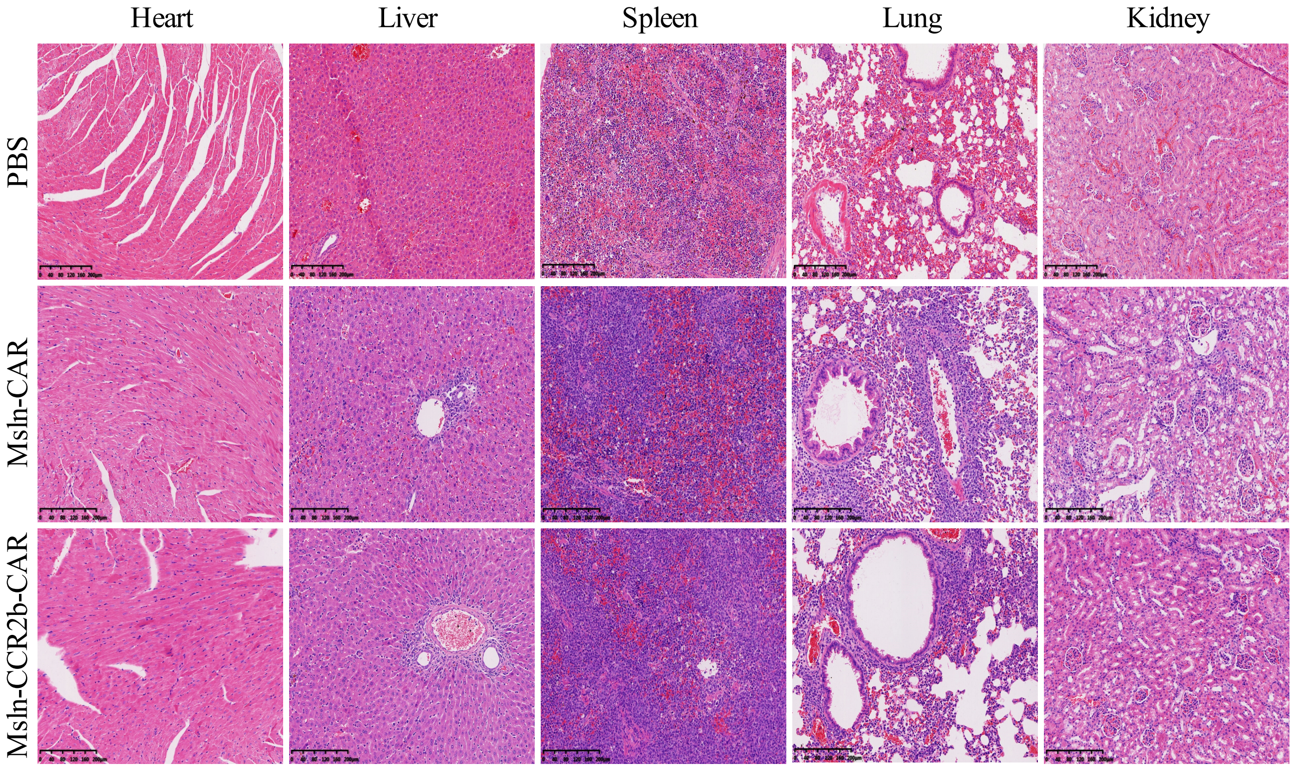

Supplementary Figure 5. Representative H&E staining of major organs (heart, liver, spleen, lung and kidney). Scale bars: 200 µm for 10×.
